# Undifferentiated embryonal sarcoma of the liver mistaken for hepatic abscess in an adult

**DOI:** 10.3892/ol.2014.2235

**Published:** 2014-06-11

**Authors:** ZI-YING XIE, LI-PING LI, WEI-JING WU, DA-YONG SUN, MEI-HUA ZHOU, YA-GANG ZHAO

**Affiliations:** 1 Department of Gastroenterology, Guangzhou General Hospital of Guangzhou Military Command, Guangzhou, Guangdong 510010, P.R. China; 2 Department of Respiratory, The Second Affiliated Hospital of Fujian Medical University, Quanzhou, Fujian 362000, P.R. China

**Keywords:** liver, undifferentiated embryonal sarcoma, misdiagnosis, hepatic abscess

## Abstract

Undifferentiated embryonal sarcoma of the liver (UESL) predominantly occurs in children under the age of 10 years, and ~90% of cases occur in children <15 years old. Patients may complain of abdominal pain, fever or other symptoms. No significant decrease has been identified in the hepatic function or elevation of α-fetoprotein, which differentiates UESL from primary carcinomas of the liver. In the present study, a rare and misdiagnosed case of an UESL arising in a male, which was mistaken for a hepatic abscess and retrospectively re-diagnosed, is reported. This case was misdiagnosed as a hepatic abscess initially, and it was diagnosed as UESL subsequent to performing tests, including a type-B ultrasonic scan and computed tomography (CT), and evaluating pathological findings. The rapid recurrence of the tumor in this patient was identified by CT, and this is associated with the malignancy of the disease. Currently, patients with UESL have a poor prognosis as there is not a successful treatment strategy. The present study analyzes the course of diagnosis and potential treatment for the disease.

## Introduction

It has been reported that undifferentiated embryonal sarcoma of the liver (UESL) occurs in children aged between six and 15 years (1,2), however, some adult cases have also been reported (3). The occurrence of UESL in patients aged ≥45 years is extremely rare, with only 27 reported cases in the English language literature up to 2012. Patients commonly complain of idiopathic upper abdominal pain, lasting for >10 days (4). The genetic aberrations of undifferentiated embryonal sarcoma (UES) are not completely understood and the misdiagnosis rate of UES is high. The most effective therapy is surgery, however, the prognosis of UES is poor. Complete surgical resection and adjuvant chemotherapy may benefit patients with UESL (5).

Currently, primary hepatic carcinoma, which was first reported by Stocker and Ishak in 1978 (2), is a common disease in the hepatopathy domain, but undifferentiated embryonal sarcoma of liver is an infrequent type of tumor with a high malignancy and peak incidence in late childhood. From 1978 to the present, there have only been ~150 cases reported (6).

The current case presents a 39-year-old male who was diagnosed with UESL, and the features surrounding the UESL and all the outcomes of this case are discussed.

## Case report

The patient was a 39-year-old male with an uncertain cause of fever and upper abdominal pain. The highest recorded temperature was 39°C and this did not return to normal on its own accord, and the upper abdominal pain was constant. The patient was previously in good health and there was no hepatitis or any particular pathography in the family and personal history. The patient was being treated at another hospital, but the treatment did not improve the symptoms and it was decided that the patient be transferred to the Guangzhou General Hospital of Guangzhou Military Command (Guangzhou, China) for further medical treatment.

**Figure 1:**
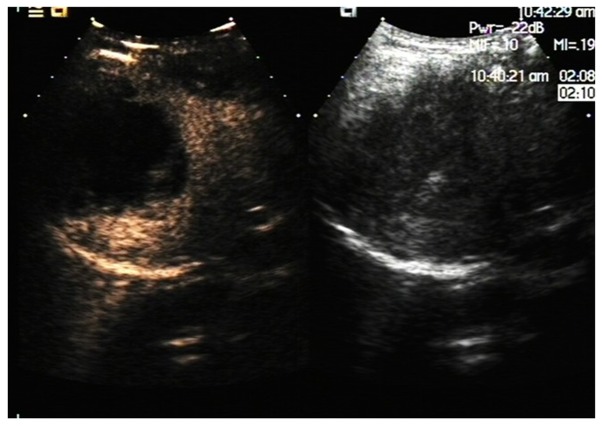
A contrast-enhanced ultrasound showing that the liver capsule is smooth, the size is normal and the internal echo is uniform. There is a 90×67 mm echo in the right liver lobe, with a clear boundary and shape. The contrast-enhanced ultrasound shows a clear boundary, circular enhancement in the arterial phase, no enhancement in the center, tantamount enhancement in the portal vein and a delayed phase.

**Figure 2:**
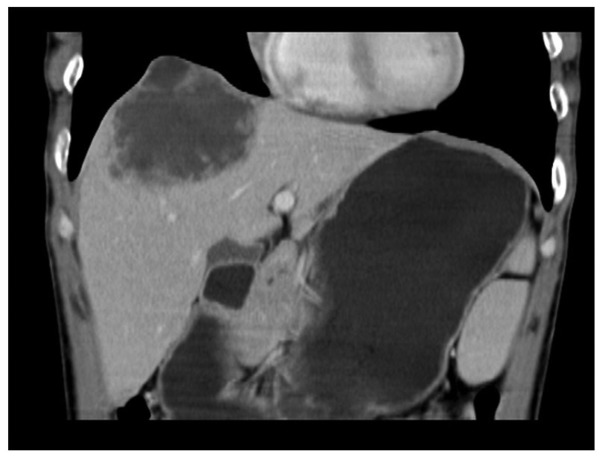
A low-density shadow in S8 of the liver, with a size of ~85×70 mm, a clear edge, an uneven density, a separate internal enhancement and no obvious solid component.

**Figure 3:**
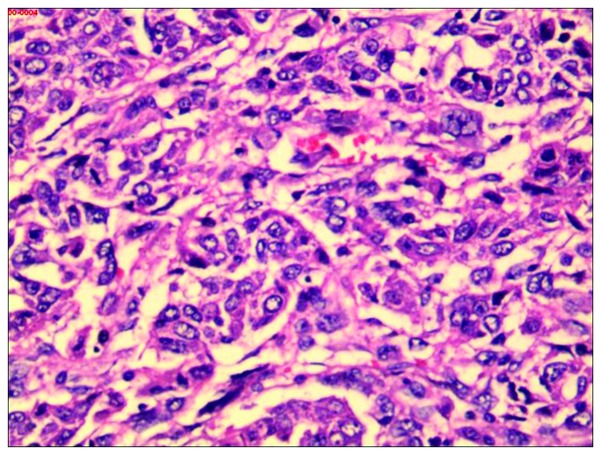
Tumor cells are shown to be polygonal or spindle in shape, with fascicula arrangement. The cells are atypical and there are numerous multi-nucleated giant cells and deformed mononuclear cells (stain, hematoxylin and eosin; magnification, ×400).

**Figure 4:**
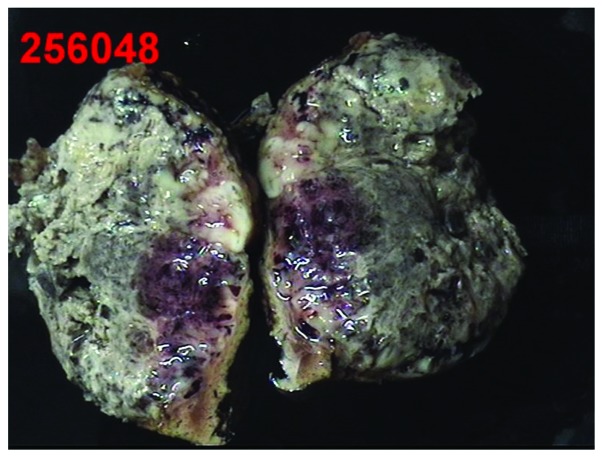
Surgical liver specimens showing the ~9.5×5.7×6.5 cm mass. The majority of the pink-grey substance is necrotic tissue in the longitudinal section.

On admission, the body temperature of the patient was 38.5°C, the pulse rate was 80 beats/min and the blood pressure was 120/78 mmHg. The patient experienced a little pain when pressure was applied to the hepatic region. There was no evidence of an underlying liver disease upon serological examination, and tumor markers, including carcinoembryonic antigen (CEA) and carbohydrate antigen (CA-199), were negative, with the exception of α-feto protein (AFP) which was present at 13.14 μg/l (range, 0–7 μg/l). Serology was also negative for hepatitis A, B, C and E, syphilis and human immunodeficiency virus. The abdominal contrast-enhanced ultrasound revealed the presence of a cystic mass (90×67 mm) in the right hepatic region with homogeneous enhancement (Fig. 1). The abdominal computed tomography (CT) scan showed a UESL of 90 mm in maximum diameter, as well as cystic lesions with a low density that was reflected as a fluid (Fig. 2). Therefore, the initial diagnosis was a hepatic abscess and anti-infective therapy (0.6 g levofloxacin intraveneously once a day and 0.5g ornidazole intraveneously twice a day, both by intradermal injection) was administered. There was no improvement following 10 days of treatment and, therefore, the patient underwent ultrasound-guided liver puncture drainage. An 18-gauge puncture needle was used and the drainage tube was placed into the abscess cavity during the procedure, but it failed to drain the pus. The histopathological analysis of the liver tissue obtained by biopsy revealed atypical, multi-nucleated giant cells and abnormal cells (Fig. 3). By immunohistochemistry, the tumor cells were positive for vimentin and α-1-antichymotrypsin; negative for cytokeratin 7 (CK7), CK19, CK8/18, hepatocyte paraffin, mucin-1, cluster of differentiation 31 (CD31), CD34 and AFP; and the positive rate of Ki67 was 80%. Therefore, a diagnosis of UESL was determined and the patient underwent liver tumor resection and diaphragmatic tumor excision surgeries. During the surgery, the liver showed moderate-diffuse nodular sclerosis and the right lobe of the tumor diaphragmatic adhesions could not be separated. The tumor ulceration is normally removed and violation of the diaphragm is also taken into consideration (Fig. 4). The patient returned to our hospital to receive regular CT examinations and, after 2 months, it was found that the tumor had recurred, as shown by the CT imaging. The patient did not accept the option of postoperative chemotherapy due to economic problems and poor knowledge of the tumor.

## Discussion

UESL is a type of rare malignant mesenchymal tumor that has the characteristics of a low incidence rate, a high degree of malignancy, high mortality and poor prognosis. In recent years, few studies have been published on UESL in the literature and the pathogenesis of UESL remains unknown. Certain studies report that gene mutations may be associated with the occurrence of UESL, but studies have shown no clear correlation between the disease and hepatitis virus infection (7–9). The present case was without hepatitis virus infection and is concordant with the previous studies.

UESL commonly occurs in the right hepatic lobe, but occurrence in the left and double lobe have been found and reported in the literature (10). The incidence of this disease has no significant difference between genders (11–13), but certain studies have indicated that the occurrence in males is slightly more common compared with that in females (14–16). In the present case, the UESL was located in the right lobe of the liver in a male. The patient may develop a fever, abdominal pain, weight loss and other non-specific symptoms, as UESL has a lack of characteristic clinical manifestations. Additionally, the disease has a lack of associated serological examination standards and medical imaging characteristics. Therefore, a definite clinical diagnosis is difficult (17). The predominant symptoms of the present case were fever and abdominal pain, and the serum liver function biomarkers, CEA and CA199, were normal. Only AFP was mildly elevated and there was no other clinical specificity identified.

A review of the associated literature has identified that UESL can be misdiagnosed as a hepatic cyst due to CT examination of a large number of UESL cases showing cystic changes, so the misdiagnosis rate is as high as 23.5%, clinically (18,19). By contrast, specific studies have indicated that the results of examination by ultrasound and CT imaging of UESL were conflicting, as the light group in ultrasound showed irregular hyperechoic, hypoechoic or mixed, but the CT has shown low-density cystic changes. Therefore, there is a certain belief that if there were variations of ultrasound and CT imaging in liver lesions, then it is necessary to take UESL into consideration. The CT imaging in the present case was similar to the early CT imaging of the hepatapostema, according to a slightly hypodense shadow, a clear edge, uneven density, no obvious parenchymatous lesion and the separate, enhanced disjunctive enhancement. There was an equi-echo display in the light echo of the liver ultrasonic images. Thus, the clinical misdiagnosis occurred. Pathological morphology and immunohistology are the most significant methods for the diagnosis of UESL and the clinical treatment effect is unsatisfactory. The liver biopsy was taken by percutaneous transhepatic cholangiography (PTC) following the failure of the liver-puncture drainage.

UESL is a type of tumor with a high degree of malignancy, fast clinical progression and unfavorable prognosis. Surgical excision is a significant way to treat UESL early. Faraj *et al* (11) indicated that the average survival rate of patients who had accepted chemotherapy or radiation therapy following surgery was higher compared with simple surgical cases. At present, there are only four child case studies on the treatment of the UESL by orthotropic liver transplantation; however, for adult treatment of UESL, using liver transplantation has been considered controversial (12,13). Only surgical treatment was performed in the present case without any further postoperative treatment, and it was indicated that tumor recurrence had occurred 2 months later by a hospital review of the CT imaging. It has been revealed that UESL is a type of infrequent liver disease with characteristics that include rapid progression and an unfavorable prognosis. For patients with liver lesions and a fever of an undetermined origin, extracting a biopsy by PTC and performing pathological morphology and immunohistology testing early is necessary.
